# 7-(4-Methyl­phen­yl)cyclo­penta­[*a*]quinolizine-10-carbaldehyde

**DOI:** 10.1107/S1600536810042467

**Published:** 2010-10-30

**Authors:** Victor B. Rybakov, Pavel V. Gormay, Eugene V. Babaev

**Affiliations:** a Department of Chemistry, Moscow State University, 119992 Moscow, Russian Federation

## Abstract

In the title compound, C_20_H_15_NO, the heterotricycle is essential planar [maximum deviation = 0.0790 (5) Å] and makes a dihedral angle of 50.70 (2)° with the benzene ring. The formyl group is almost coplanar with the tricyclic ring, the C—C—C—O torsion angle being −0.78 (13)°.

## Related literature

For background to the Vilsmeier–Haack reaction, see: Laue & Plagens (2005 ▶). For a related structure, see: Borisenko *et al.* (1996 ▶).
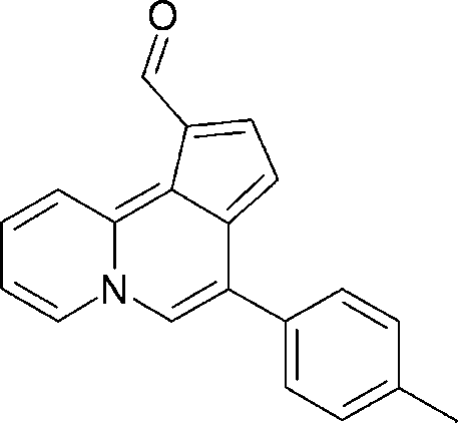


## Experimental

### 

#### Crystal data

C_20_H_15_NO
*M* *_r_* = 285.33
Triclinic, 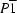

*a* = 7.2907 (13) Å
*b* = 8.9627 (14) Å
*c* = 12.0162 (19) Å
α = 88.48 (2)°
β = 81.400 (19)°
γ = 67.821 (18)°
*V* = 718.5 (2) Å^3^
*Z* = 2
Cu *K*α radiation
μ = 0.64 mm^−1^
*T* = 295 K
0.15 × 0.13 × 0.11 mm

#### Data collection

Enraf–Nonius CAD-4 diffractometer
Absorption correction: refined from Δ*F* (Walker & Stuart, 1983 ▶) *T*_min_ = 0.649, *T*_max_ = 1.000
3186 measured reflections
2909 independent reflections
2394 reflections with *I* > 2σ(*I*)
*R*_int_ = 0.000 please give correct value
1 standard reflections every 60 min  intensity decay: 5%

#### Refinement

*R*[*F* ^2^ > 2σ(*F* ^2^)] = 0.024
*wR*(*F* ^2^) = 0.056
*S* = 0.96
2909 reflections
200 parameters
61 restraints
H-atom parameters constrained
Δρ_max_ = 0.08 e Å^−3^
Δρ_min_ = −0.10 e Å^−3^

### 

Data collection: *CAD-4 EXPRESS* (Enraf–Nonius, 1994 ▶); cell refinement: *CAD-4 EXPRESS*; data reduction: *XCAD4* (Harms & Wocadlo, 1995 ▶); program(s) used to solve structure: *SHELXS97* (Sheldrick, 2008 ▶); program(s) used to refine structure: *SHELXL97* (Sheldrick, 2008 ▶); molecular graphics: *ORTEP-3* (Farrugia, 1997 ▶); software used to prepare material for publication: *WinGX* (Farrugia, 1999 ▶).

## Supplementary Material

Crystal structure: contains datablocks global, I. DOI: 10.1107/S1600536810042467/wm2411sup1.cif

Structure factors: contains datablocks I. DOI: 10.1107/S1600536810042467/wm2411Isup2.hkl

Additional supplementary materials:  crystallographic information; 3D view; checkCIF report

## Figures and Tables

**Fig. 1.**
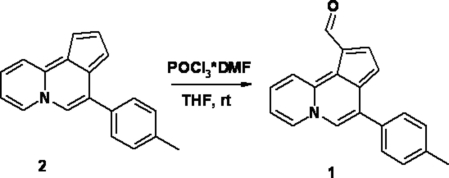
Synthesis of the title compound.

**Fig. 2.**
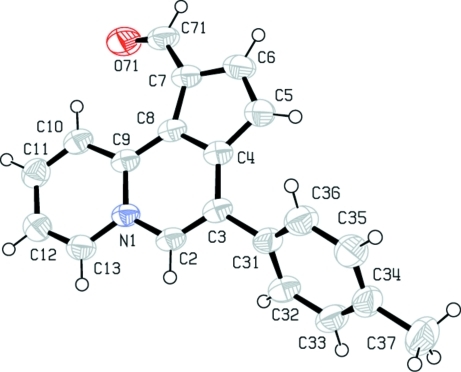
ORTEP-3 plot of the molecular structure of the title compound showing the atom-numbering scheme. Displacement ellipsoids are drawn at the 50% probability level. H atoms are presented as small spheres of arbitrary radius.

## supplementary crystallographic information

### Comment

Cyclopenta[*a*]quinolizines are a novel subclass of non-benzenoid heterocycles π-isoelectronic with azulene, so called pseudoazulenes. Some pseudoazulenes show ambident reactivity towards electrophiles, since both α-sites of the cyclopentadiene ring can be substituted. The Vilsmeier–Haack reaction (Laue & Plagens, 2005) (Fig. 1) was one of the simplest tests to estimate the reactivity of cyclopenta[*a*]quinolizines and the regioselectivity of substitution.

We found that only one product was formed in the reaction. Simple ^1^H NMR spectra cannot provide an unambiguous proof of the site of substitution. By *X*-ray analysis we proved that the product is the title compound. From this viewpoint it becomes evident, that the strong shift of the proton H-4 signal (10.53 p.p.m. in 1 against 8.16 in the initial compound 2; Fig. 1) observed in ^1^H NMR spectra is caused by the *peri*-effect of the formyl group at C7.

In the title compound 1 (Fig. 2), the bond lengths in the heterocyclic core show slight alternations. The bond length between C7 and C71 of the carbonyl group (1.4351 (8) Å) is much shorter than that in the structure of the simplest aromatic ketone, benzaldehyde (1.477 (3) Å; Borisenko *et al.*, 1996). Since the formyl group is almost co-planar with the tricyclic ring (the torsion angle C8—C7—C71═O71 is -0.78 (13)°), it may indicate strong conjugation of the carbonyl group with the π-excessive cyclopentadiene ring.

### Experimental

Freshly distilled DMF (1 ml) was added at 263 K to the solution of POCl_3_ (2.34 mmol, 357 mg) in dry THF (15 ml) forming the Vilsmeier reagent. The solution of 7-(4-methylphenyl)cyclopenta[*a*]quinolizine 2 (300 mg, 1.17 mmol) in dry THF (10 ml) was added dropwise at 273 K to the Vilsmeier reagent. The mixture was stirred overnight at room temperature, diluted with water, and neutralized by NaOH to pH≈ 8. The resultant precipitate was filtered off and recrystallized from DMF. Yield of 1: 311 mg (93%), m.p. = 527–528 K.

^1^H NMR (400 MHz; CDCl_3_; δ, p.p.m.; J, Hz): 2.47 (s, 3H, CH_3_), 6.80 (d, J = 4.0, 1H), 7.12 (m, 1H), 7.35 (m, 2H, *Ar*H), 7.61 (m, 2H, *Ar*H), 7.64 (s, 1H), 7.77 (d, J = 4.0, 1H), 7.82 (s, 1H), 8.21 (d, J = 7.1, 1H, H4), 9.92 (s, 1H, CHO), 10.53 (d, J = 7.1, 1H, H1).

### Refinement

C-bound H-atoms were placed in calculated positions (C—H 0.93Å; 0.96 Å) and refined as riding, with *U*_iso_(H) = 1.2(1.5)*U*_eq_(C). The initial experimental data were measured for a full sphere, but at the final stage of the refinement, the 'MERG 2' instruction was used in *SHELXL* and the *DIFABS* procedure (Walker & Stuart, 1983) was applied. As a result, we have FVAR = 1, *R*_int_ = 0, and the experimental data were reduced to a half-sphere with indices -8 ≤*h*≤ +8, -10 ≤*k*≤ +11 and 0 ≤*l*≤ +15.

### Crystal data

**Table:**

C_20_H_15_NO	*Z* = 2
*M**_r_* = 285.33	*F*(000) = 300
Triclinic, *P*1	*D*_x_ = 1.319 Mg m^−^^3^
Hall symbol: -P 1	Melting point = 527–528 K
*a* = 7.2907 (13) Å	Cu *K*α radiation, λ = 1.54184 Å
*b* = 8.9627 (14) Å	Cell parameters from 25 reflections
*c* = 12.0162 (19) Å	θ = 32.0–34.9°
α = 88.48 (2)°	µ = 0.64 mm^−^^1^
β = 81.400 (19)°	*T* = 295 K
γ = 67.821 (18)°	Prism, pale yellow
*V* = 718.5 (2) Å^3^	0.15 × 0.13 × 0.11 mm

### Data collection

**Table:**

Enraf–Nonius CAD-4 diffractometer	2394 reflections with *I* > 2σ(*I*)
Radiation source: fine-focus sealed tube	*R*_int_ = 0.0000
graphite	θ_max_ = 75.2°, θ_min_ = 3.7°
non–profiled ω scans	*h* = −8→9
Absorption correction: part of the refinement model (Δ*F*) (Walker & Stuart, 1983)	*k* = −10→11
*T*_min_ = 0.649, *T*_max_ = 1.000	*l* = −11→15
3186 measured reflections	1 standard reflections every 60 min
2909 independent reflections	intensity decay: 5%

### Refinement

**Table:**

Refinement on *F*^2^	Primary atom site location: structure-invariant direct methods
Least-squares matrix: full	Secondary atom site location: difference Fourier map
*R*[*F*^2^ > 2σ(*F*^2^)] = 0.024	Hydrogen site location: inferred from neighbouring sites
*wR*(*F*^2^) = 0.056	H-atom parameters constrained
*S* = 0.96	*w* = 1/[σ^2^(*F*_o_^2^) + (0.0398*P*)^2^] where *P* = (*F*_o_^2^ + 2*F*_c_^2^)/3
2909 reflections	(Δ/σ)_max_ = 0.001
200 parameters	Δρ_max_ = 0.08 e Å^−^^3^
61 restraints	Δρ_min_ = −0.10 e Å^−^^3^

### Special details

**Table:**

Geometry. All s.u.'s (except the s.u. in the dihedral angle between two l.s. planes) are estimated using the full covariance matrix. The cell s.u.'s are taken into account individually in the estimation of s.u.'s in distances, angles and torsion angles; correlations between s.u.'s in cell parameters are only used when they are defined by crystal symmetry. An approximate (isotropic) treatment of cell s.u.'s is used for estimating s.u.'s involving l.s. planes.
Refinement. Refinement of *F*^2^ against ALL reflections. The weighted *R*-factor *wR* and goodness of fit *S* are based on *F*^2^, conventional *R*-factors *R* are based on *F*, with *F* set to zero for negative *F*^2^. The threshold expression of *F*^2^ > 2σ(*F*^2^) is used only for calculating *R*-factors(gt) *etc*. and is not relevant to the choice of reflections for refinement. *R*-factors based on *F*^2^ are statistically about twice as large as those based on *F*, and *R*-factors based on ALL data will be even larger.

### Fractional atomic coordinates and isotropic or equivalent isotropic displacement parameters (Å^2^)

**Table:**

	*x*	*y*	*z*	*U*_iso_*/*U*_eq_	
N1	0.20346 (7)	0.58547 (5)	0.01802 (3)	0.05065 (12)	
C2	0.09520 (9)	0.71381 (6)	0.09351 (4)	0.05806 (16)	
H2	0.0799	0.8176	0.0716	0.070*	
C3	0.01070 (9)	0.69255 (6)	0.19859 (4)	0.05301 (14)	
C31	−0.11306 (9)	0.83714 (6)	0.27192 (4)	0.05427 (14)	
C32	−0.25884 (9)	0.96616 (6)	0.22956 (5)	0.06118 (16)	
H32	−0.2771	0.9621	0.1549	0.073*	
C33	−0.37705 (9)	1.10046 (6)	0.29708 (5)	0.06566 (17)	
H33	−0.4751	1.1849	0.2674	0.079*	
C34	−0.35215 (9)	1.11184 (7)	0.40883 (5)	0.06390 (17)	
C35	−0.20805 (10)	0.98237 (7)	0.45021 (5)	0.06909 (18)	
H35	−0.1897	0.9867	0.5248	0.083*	
C36	−0.08989 (9)	0.84623 (7)	0.38403 (4)	0.06206 (16)	
H36	0.0053	0.7606	0.4146	0.074*	
C37	−0.47854 (12)	1.25985 (8)	0.48145 (6)	0.0930 (3)	
H37A	−0.4965	1.2300	0.5584	0.140*	
H37B	−0.6068	1.3088	0.4568	0.140*	
H37C	−0.4128	1.3352	0.4755	0.140*	
C4	0.03745 (9)	0.53309 (6)	0.23109 (4)	0.05122 (14)	
C5	−0.03475 (10)	0.47207 (7)	0.33034 (5)	0.06394 (17)	
H5	−0.1069	0.5315	0.3957	0.077*	
C6	0.02069 (10)	0.30912 (7)	0.31294 (5)	0.06486 (17)	
H6	−0.0075	0.2406	0.3662	0.078*	
C7	0.12585 (9)	0.25977 (6)	0.20378 (4)	0.05555 (15)	
C71	0.17458 (10)	0.09745 (6)	0.16468 (5)	0.06569 (17)	
H71	0.1522	0.0286	0.2196	0.079*	
O71	0.24109 (8)	0.03569 (5)	0.07002 (4)	0.08139 (15)	
C8	0.13764 (8)	0.40156 (6)	0.15092 (4)	0.05205 (14)	
C9	0.23175 (8)	0.42748 (5)	0.04415 (4)	0.04982 (14)	
C10	0.35065 (9)	0.30555 (6)	−0.03740 (4)	0.05922 (16)	
H10	0.3721	0.1986	−0.0218	0.071*	
C11	0.43433 (9)	0.34090 (7)	−0.13810 (5)	0.06062 (16)	
H11	0.5140	0.2589	−0.1903	0.073*	
C12	0.39948 (9)	0.50275 (7)	−0.16278 (5)	0.06187 (16)	
H12	0.4525	0.5283	−0.2326	0.074*	
C13	0.28977 (9)	0.62042 (7)	−0.08572 (4)	0.05829 (16)	
H13	0.2711	0.7268	−0.1018	0.070*	

### Atomic displacement parameters (Å^2^)

**Table:**

	*U*^11^	*U*^22^	*U*^33^	*U*^12^	*U*^13^	*U*^23^
N1	0.0615 (3)	0.03132 (19)	0.0522 (2)	−0.01155 (18)	−0.00431 (18)	0.00552 (15)
C2	0.0769 (4)	0.0302 (2)	0.0558 (3)	−0.0096 (2)	−0.0049 (2)	0.00297 (18)
C3	0.0637 (4)	0.0335 (2)	0.0543 (3)	−0.0101 (2)	−0.0086 (2)	0.00249 (18)
C31	0.0664 (4)	0.0342 (2)	0.0574 (3)	−0.0167 (2)	−0.0006 (2)	−0.00026 (19)
C32	0.0758 (4)	0.0377 (2)	0.0636 (3)	−0.0150 (2)	−0.0081 (3)	0.0018 (2)
C33	0.0694 (4)	0.0375 (3)	0.0808 (3)	−0.0125 (2)	−0.0032 (3)	−0.0015 (2)
C34	0.0673 (4)	0.0436 (3)	0.0761 (3)	−0.0236 (3)	0.0126 (3)	−0.0100 (2)
C35	0.0923 (5)	0.0540 (3)	0.0574 (3)	−0.0280 (3)	0.0017 (3)	−0.0071 (2)
C36	0.0737 (4)	0.0468 (3)	0.0595 (3)	−0.0159 (3)	−0.0095 (3)	−0.0008 (2)
C37	0.1015 (6)	0.0583 (4)	0.1010 (5)	−0.0230 (4)	0.0258 (4)	−0.0257 (3)
C4	0.0603 (3)	0.0355 (2)	0.0534 (2)	−0.0133 (2)	−0.0083 (2)	0.00462 (18)
C5	0.0812 (4)	0.0469 (3)	0.0529 (3)	−0.0149 (3)	−0.0034 (2)	0.0080 (2)
C6	0.0814 (4)	0.0456 (3)	0.0606 (3)	−0.0183 (3)	−0.0076 (3)	0.0167 (2)
C7	0.0665 (4)	0.0346 (2)	0.0603 (3)	−0.0136 (2)	−0.0102 (2)	0.01082 (19)
C71	0.0786 (4)	0.0343 (2)	0.0753 (3)	−0.0135 (2)	−0.0078 (3)	0.0114 (2)
O71	0.1109 (4)	0.0401 (2)	0.0856 (3)	−0.0254 (2)	0.0002 (3)	−0.00102 (19)
C8	0.0609 (3)	0.0335 (2)	0.0536 (2)	−0.0094 (2)	−0.0077 (2)	0.00764 (18)
C9	0.0600 (3)	0.0315 (2)	0.0536 (2)	−0.0124 (2)	−0.0089 (2)	0.00413 (18)
C10	0.0743 (4)	0.0354 (2)	0.0592 (3)	−0.0124 (2)	−0.0055 (2)	−0.0013 (2)
C11	0.0644 (4)	0.0505 (3)	0.0600 (3)	−0.0159 (3)	−0.0026 (2)	−0.0062 (2)
C12	0.0715 (4)	0.0561 (3)	0.0520 (3)	−0.0203 (3)	−0.0017 (2)	0.0028 (2)
C13	0.0727 (4)	0.0431 (3)	0.0548 (3)	−0.0195 (2)	−0.0045 (2)	0.0091 (2)

### Geometric parameters (Å, °)

**Table:**

N1—C9	1.3862 (7)	C37—H37C	0.9600
N1—C2	1.3873 (7)	C4—C5	1.4119 (8)
N1—C13	1.3934 (7)	C4—C8	1.4321 (7)
C2—C3	1.3614 (8)	C5—C6	1.3731 (8)
C2—H2	0.9300	C5—H5	0.9300
C3—C4	1.4198 (7)	C6—C7	1.4064 (8)
C3—C31	1.4865 (8)	C6—H6	0.9300
C31—C32	1.3887 (8)	C7—C8	1.4315 (8)
C31—C36	1.3909 (8)	C7—C71	1.4351 (8)
C32—C33	1.3818 (8)	C71—O71	1.2252 (7)
C32—H32	0.9300	C71—H71	0.9300
C33—C34	1.3930 (9)	C8—C9	1.4185 (8)
C33—H33	0.9300	C9—C10	1.4119 (7)
C34—C35	1.3794 (9)	C10—C11	1.3570 (8)
C34—C37	1.5061 (8)	C10—H10	0.9300
C35—C36	1.3843 (8)	C11—C12	1.4065 (9)
C35—H35	0.9300	C11—H11	0.9300
C36—H36	0.9300	C12—C13	1.3428 (8)
C37—H37A	0.9600	C12—H12	0.9300
C37—H37B	0.9600	C13—H13	0.9300
			
C9—N1—C2	122.34 (5)	C5—C4—C3	132.01 (5)
C9—N1—C13	120.37 (5)	C5—C4—C8	107.93 (5)
C2—N1—C13	117.25 (5)	C3—C4—C8	119.75 (5)
C3—C2—N1	122.08 (5)	C6—C5—C4	107.68 (5)
C3—C2—H2	119.0	C6—C5—H5	126.2
N1—C2—H2	119.0	C4—C5—H5	126.2
C2—C3—C4	118.24 (5)	C5—C6—C7	110.93 (6)
C2—C3—C31	118.72 (5)	C5—C6—H6	124.5
C4—C3—C31	122.97 (5)	C7—C6—H6	124.5
C32—C31—C36	118.33 (5)	C6—C7—C8	106.27 (5)
C32—C31—C3	120.04 (5)	C6—C7—C71	119.19 (6)
C36—C31—C3	121.61 (5)	C8—C7—C71	134.02 (5)
C33—C32—C31	120.75 (6)	O71—C71—C7	129.90 (6)
C33—C32—H32	119.6	O71—C71—H71	115.1
C31—C32—H32	119.6	C7—C71—H71	115.1
C32—C33—C34	121.27 (6)	C9—C8—C7	132.70 (5)
C32—C33—H33	119.4	C9—C8—C4	120.05 (5)
C34—C33—H33	119.4	C7—C8—C4	107.18 (5)
C35—C34—C33	117.44 (5)	N1—C9—C10	117.65 (5)
C35—C34—C37	121.47 (6)	N1—C9—C8	117.11 (5)
C33—C34—C37	121.09 (6)	C10—C9—C8	125.24 (5)
C34—C35—C36	122.00 (6)	C11—C10—C9	121.49 (5)
C34—C35—H35	119.0	C11—C10—H10	119.3
C36—C35—H35	119.0	C9—C10—H10	119.3
C35—C36—C31	120.20 (6)	C10—C11—C12	119.40 (5)
C35—C36—H36	119.9	C10—C11—H11	120.3
C31—C36—H36	119.9	C12—C11—H11	120.3
C34—C37—H37A	109.5	C13—C12—C11	120.09 (5)
C34—C37—H37B	109.5	C13—C12—H12	120.0
H37A—C37—H37B	109.5	C11—C12—H12	120.0
C34—C37—H37C	109.5	C12—C13—N1	120.95 (5)
H37A—C37—H37C	109.5	C12—C13—H13	119.5
H37B—C37—H37C	109.5	N1—C13—H13	119.5
			
C9—N1—C2—C3	0.42 (9)	C5—C6—C7—C71	−172.26 (6)
C13—N1—C2—C3	−177.64 (5)	C6—C7—C71—O71	169.69 (7)
N1—C2—C3—C4	0.70 (9)	C8—C7—C71—O71	−0.78 (13)
N1—C2—C3—C31	−176.42 (5)	C6—C7—C8—C9	176.75 (6)
C2—C3—C31—C32	47.59 (9)	C71—C7—C8—C9	−11.90 (12)
C4—C3—C31—C32	−129.39 (7)	C6—C7—C8—C4	−0.13 (7)
C2—C3—C31—C36	−133.71 (7)	C71—C7—C8—C4	171.22 (7)
C4—C3—C31—C36	49.31 (9)	C5—C4—C8—C9	−177.74 (5)
C36—C31—C32—C33	0.33 (10)	C3—C4—C8—C9	7.92 (9)
C3—C31—C32—C33	179.07 (5)	C5—C4—C8—C7	−0.38 (7)
C31—C32—C33—C34	0.92 (10)	C3—C4—C8—C7	−174.73 (6)
C32—C33—C34—C35	−1.43 (10)	C2—N1—C9—C10	−177.72 (5)
C32—C33—C34—C37	178.76 (6)	C13—N1—C9—C10	0.29 (8)
C33—C34—C35—C36	0.72 (10)	C2—N1—C9—C8	2.57 (8)
C37—C34—C35—C36	−179.47 (6)	C13—N1—C9—C8	−179.42 (5)
C34—C35—C36—C31	0.51 (10)	C7—C8—C9—N1	176.80 (6)
C32—C31—C36—C35	−1.03 (9)	C4—C8—C9—N1	−6.65 (8)
C3—C31—C36—C35	−179.75 (6)	C7—C8—C9—C10	−2.89 (11)
C2—C3—C4—C5	−177.57 (6)	C4—C8—C9—C10	173.67 (5)
C31—C3—C4—C5	−0.58 (11)	N1—C9—C10—C11	−0.16 (9)
C2—C3—C4—C8	−4.81 (9)	C8—C9—C10—C11	179.52 (5)
C31—C3—C4—C8	172.18 (5)	C9—C10—C11—C12	−1.05 (10)
C3—C4—C5—C6	174.15 (7)	C10—C11—C12—C13	2.19 (10)
C8—C4—C5—C6	0.76 (7)	C11—C12—C13—N1	−2.10 (10)
C4—C5—C6—C7	−0.87 (8)	C9—N1—C13—C12	0.86 (9)
C5—C6—C7—C8	0.62 (7)	C2—N1—C13—C12	178.96 (5)
